# The AI gambit: leveraging artificial intelligence to combat climate change—opportunities, challenges, and recommendations

**DOI:** 10.1007/s00146-021-01294-x

**Published:** 2021-10-18

**Authors:** Josh Cowls, Andreas Tsamados, Mariarosaria Taddeo, Luciano Floridi

**Affiliations:** 1 Oxford Internet Institute, University of Oxford, 1 St Giles’, Oxford, OX1 3JS UK; 2 Alan Turing Institute, British Library, 96 Euston Rd, London, NW1 2DB UK

**Keywords:** Artificial intelligence, Climate change, Digital ethics, Digital governance, Environment, Sustainability, Carbon footprint

## Abstract

In this article, we analyse the role that artificial intelligence (AI) could play, and is playing, to combat global climate change. We identify two crucial opportunities that AI offers in this domain: it can help improve and expand current understanding of climate change, and it can contribute to combatting the climate crisis effectively. However, the development of AI also raises two sets of problems when considering climate change: the possible exacerbation of social and ethical challenges already associated with AI, and the contribution to climate change of the greenhouse gases emitted by training data and computation-intensive AI systems. We assess the carbon footprint of AI research, and the factors that influence AI’s greenhouse gas (GHG) emissions in this domain. We find that the carbon footprint of AI research may be significant and highlight the need for more evidence concerning the trade-off between the GHG emissions generated by AI research and the energy and resource efficiency gains that AI can offer. In light of our analysis, we argue that leveraging the opportunities offered by AI for global climate change whilst limiting its risks is a gambit which requires responsive, evidence-based, and effective governance to become a winning strategy. We conclude by identifying the European Union as being especially well-placed to play a leading role in this policy response and provide 13 recommendations that are designed to identify and harness the opportunities of AI for combatting climate change, while reducing its impact on the environment.

## Introduction

In this article, we analyse the role that artificial intelligence (AI) could play, and is already playing, as a technology to combat global climate change. The Intergovernmental Panel on Climate Change (IPCC) has repeatedly emphasised the need for large-scale responses to human-induced climate change to prevent avoidable warming and to mitigate the effects of unavoidable warming as well as that which has already occurred (Masson-Delmotte et al. 2018; Pachauri et al. 2014).

Leveraging the opportunities offered by AI for global climate change is both feasible and desirable, but it involves a sacrifice (ethical risks and potentially an increased carbon footprint) in view of a very significant gain (a more effective response to climate change). It is, in other words, a gambit, which requires responsive and effective governance to become a winning strategy. The overall aim of this article is to contribute to the development of such a strategy. We begin in Sect. 2 by exploring the opportunities that AI affords for combatting climate change, identifying two crucial opportunities: AI can help improve and expand current understanding of climate change; and AI is increasingly part of a package of responses that are essential to combatting the climate crisis effectively, by delivering much greener, more sustainable and effective solutions. However, as we argue, the introduction of AI into the climate domain risks amplifying several social and ethical challenges already associated with AI more generally, such as unfair bias, discrimination, or opacity in decision-making.

In Sect. 3, we address the flipside of AI in the context of climate change: the contribution to global climate change of the greenhouse gases emitted by developing computation-intensive AI systems. We focus on the carbon footprint of AI research, and assess the factors that influence AI’s greenhouse gas (GHG) emissions in this context, finding that the carbon footprint of AI research can be significant, and highlighting the need for more scientific evidence concerning the trade-off between the GHG emissions generated by AI research and the energy and resource efficiency gains that AI offers when applied to various tasks and industries.

In Sect. 4, we turn to the wider policy context, and identify the European Union as being especially well placed to adopt effective policy response to the opportunities and challenges presented. Thus, in Sect. 5, we provide 13 recommendations for European policymakers and AI researchers that are designed to identify and harness the opportunities of AI for combatting climate change, while reducing the impact of its development on the environment. We conclude our analysis by stressing that risks and benefits of the uses of AI to fight climate change are distinct yet intertwined, and that effective policies and strategies are required to both leverage the potential of AI and minimise the harms it poses to protect the environment.

## AI against climate change

AI is already having a significant positive impact in the fight against climate change. Yet exactly how significant, and precisely what sort of impact, are challenging questions to answer. This section provides an overview of initiatives and projects that rely on AI to understand and combat climate change (1.1), notes work already done to document this potential positive impact of AI on climate change (1.2), and identifies a set of obstacles to be overcome to ensure that the use of AI to understand and combat climate change is not only effective but also ethically sound (1.3).

### How AI is used against climate change

AI may be characterised as a set of multipurpose tools and techniques designed to simulate and/or improve upon processes that would have seemed intelligent had a human performed them (McCarthy et al. 2006). At a high level, key cognitive capabilities displayed by “intelligent” machine systems include a combination of classification, prediction, and decision-making. These capabilities are already being deployed in a diverse array of domains, like health (e.g., recognising features in an image such as an X-ray scan for cancer diagnosis), transportation (e.g., using environmental sensors to safely drive a car), and communication (e.g., processing human speech and responding in kind). Applying the “solution space” of AI to the “problem space” of climate change could yield significant benefits, by, first, helping to understand the problem, and second, by facilitating effective responses.

First, despite scientific consensus about the basic facts of climate change, many aspects of the environmental crisis remain uncertain. This includes the explanation of past and present events and observations, and the accurate prediction of future outcomes. The ability of AI to process enormous amounts of non-structured, multi-dimensional data using sophisticated optimisation techniques is already facilitating the understanding of high-dimensional climate datasets and forecasting of future trends (Huntingford et al. 2019). AI techniques have been used to forecast global mean temperature changes (Ise and Oba 2019; Cifuentes et al. 2020); predict climactic and oceanic phenomena such as El Niño (Ham et al. 2019), cloud systems (Rasp et al. 2018), and tropical instability waves (Zheng et al. 2020); better understand aspects of the weather system—like rainfall, generally (Sønderby et al. 2020; Larraondo et al. 2020) and in specific locales, such as Malaysia (Ridwan et al. 2020)—and their knock-on consequences, like water demand (Shrestha et al. 2020; Xenochristou et al. 2020). AI tools can also help anticipate the extreme weather events that are more common as a result of global climate change, for example heavy rain damage (Choi et al. 2018) and wildfires (Jaafari et al. 2019), and other downstream consequences, such as patterns of human migration (Robinson and Dilkina 2018). In many cases, AI techniques can help to improve or expedite existing forecasting and prediction systems, for example by automatically labelling climate modelling data (Chattopadhyay et al. 2020), improving approximations for simulating the atmosphere (Gagne et al. 2020), and separating signals from noise in climate observations (Barnes et al. 2019).

Second, combating climate change effectively requires a vast array of responses to the crisis, which broadly include both mitigating existing effects of climate change and reducing emissions through decarbonisation to prevent further warming. For example, a 2018 Microsoft/PwC report estimated that using AI for environmental applications could boost global GDP by between 3.1 and 4.4%, while reducing greenhouse gas emissions anywhere from 1.5 to 4% by 2030 compared to a “business as usual” scenario (Microsoft 2018, 8). An array of AI-based techniques already plays a key role in many of these responses (Inderwildi et al. 2020; Sayed-Mouchaweh 2020). This includes, for example, energy efficiency in industry, especially the petrochemical sector (Narciso and Martins 2020). Studies have also used AI to understand, at a high level, industrial pollution in China (Zhou et al. 2016), the carbon footprint of concrete used in construction (Thilakarathna et al. 2020), and even energy efficiency in shipping (Perera et al. 2016). Other work has explored the use of AI in electrical grid management (Di Piazza et al. 2020), to forecast building energy usage (Fathi et al. 2020), and to assess the sustainability of food consumption (Abdella et al. 2020). Many of these studies involve showing the potential applicability of AI-based methods in silico and/or at a small scale. However, the techniques presented could have considerable impact across society and the global economy if taken forward and scaled up.

There are also examples where AI-based approaches can help improve the understanding of, and facilitate effective responses to, climate change—particularly in the policy-making domain. For example, AI can help to predict carbon emissions based on present trends (Mardani et al. 2020; Wei et al. 2018), and help monitor the active removal of carbon from the atmosphere through sequestration (Menad et al. 2019). AI approaches have also been employed to assess the potential viability and impact of large-scale policy changes and other societal shifts. This includes top-down policy initiatives, such as carbon tax schemes (Abrell et al. 2019) and carbon trading systems (Lu et al. 2020), as well as detecting (Xiao et al. 2019) and weighing the variables associated with different travel modes (Hagenauer and Helbich 2017), and optimising electric vehicle sharing (Miao et al. 2019) and charging architecture (Tao et al. 2018). Each of these could in turn boost the availability and uptake of more climate-friendly transportation options.

Beyond this indicative evidence, the growing use of AI to fight climate change can also be seen from the higher vantage point of major institutions and large-scale initiatives. The European Lab for Learning & Intelligent Systems (ELLIS) has a Machine Learning for Earth and Climate Sciences programme that aims to “model and understand the Earth system with Machine Learning and Process Understanding”.^1^ The European Space Agency has also established a Digital Twin Earth Challenge to provide “forecasting on the impact of climate change and responding to societal challenges”.^2^ On the academic side, the EC-funded iMIRACLI (innovative MachIne leaRning to constrain Aerosol-cloud CLimate Impacts) initiative will support 15 PhD students across nine European universities to “develop machine learning solutions to deliver a breakthrough in climate research”,^3^ with doctoral projects underway since autumn 2020.

**Fig. 1:**
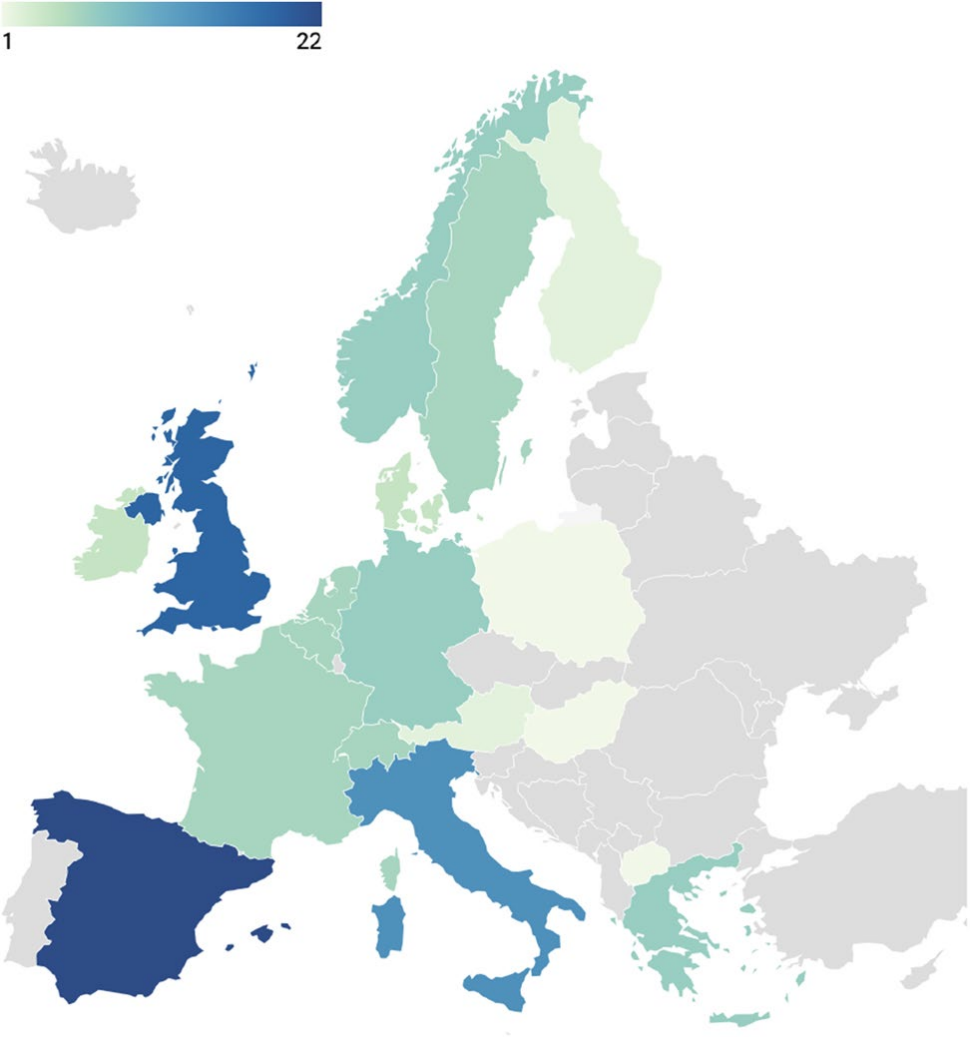
Countries in which EU-funded projects using AI to address climate change are “coordinated”. Not shown: Israel (1 project)

**Fig. 2:**
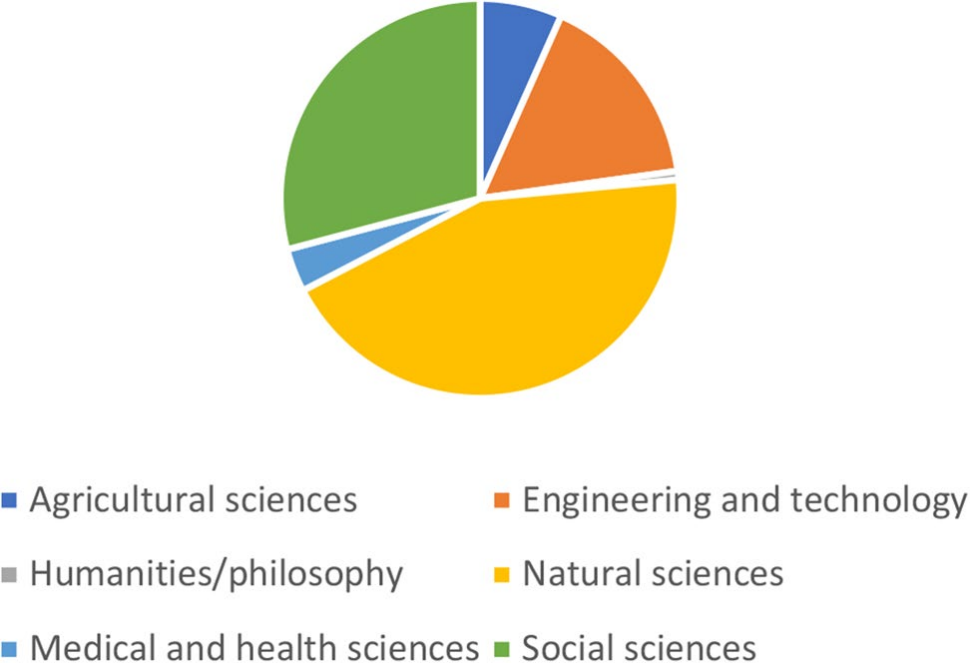
Top-level disciplinary focus of EU-funded projects using AI to address climate change

**Fig. 3:**
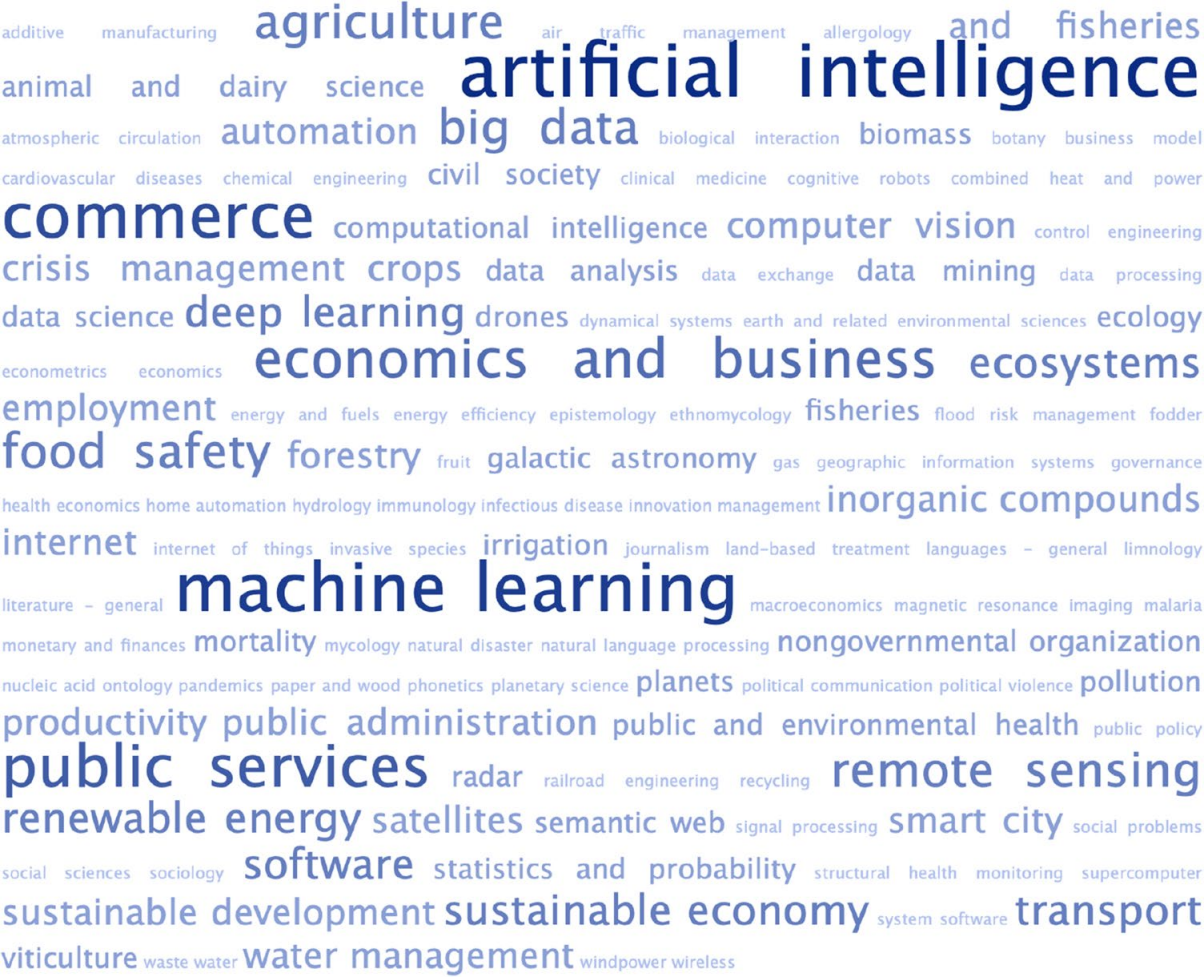
Frequency-based word cloud showing self-identified domains of EU-funded projects using AI to address climate change

Several European universities have initiatives and training programmes dedicated to unlocking the power of AI for climate.^4^^,^^5^^,^^6^ Indeed, a search of Cordis—the European database for funded research—for current projects addressing climate change and AI returned a total of 122 results.^7^ Analysis of these 122 projects suggests that they represent both geographic and disciplinary breadth. The projects are well spread across the continent, albeit with a clear skew towards western Europe in terms of where they are coordinated (see Fig. 1). Figure 2 displays the top-level field(s) of study indicated for each of the projects, where this information was provided (*n* = 106). Unsurprisingly, a large majority of projects relate to the natural sciences and/or engineering and technology, but a considerable number are also anchored in social sciences. And as Fig. 3 shows, at a more granular level, the breadth of subjects that these projects touch on is vast, and spans domains as diverse as viticulture, mycology and galactic astronomy.

There is also considerable evidence of private and non-profit initiatives using AI to combat climate change around the world. Microsoft’s AI for Earth is a 5-year $50 million initiative established in 2017, designed to support organisations and researchers using AI and other computational techniques to tackle various aspects of the climate crisis. It currently has 16 partner organisations^8^ and has released relevant open-source tools^9^ and provided grants in the form of cloud computing credits to projects using AI for a variety of purposes, from monitoring climate change in the Antarctic to protecting bird populations after hurricanes. Google’s AI for Social Good programme supports 20 organisations using AI to pursuing various socially beneficial goals with funding and cloud computing credits, including projects seeking to minimise crop damage in India, better manage waste in Indonesia, protect rainforests in the US, and improve air quality in Uganda.^10^ Meanwhile, AI development company ElementAI’s AI for Climate program^11^ provides expertise and partnership opportunities to improve the energy efficiency of manufacturing and business operations.

### How evidence of AI against climate change is gathered

**Fig. 4:**
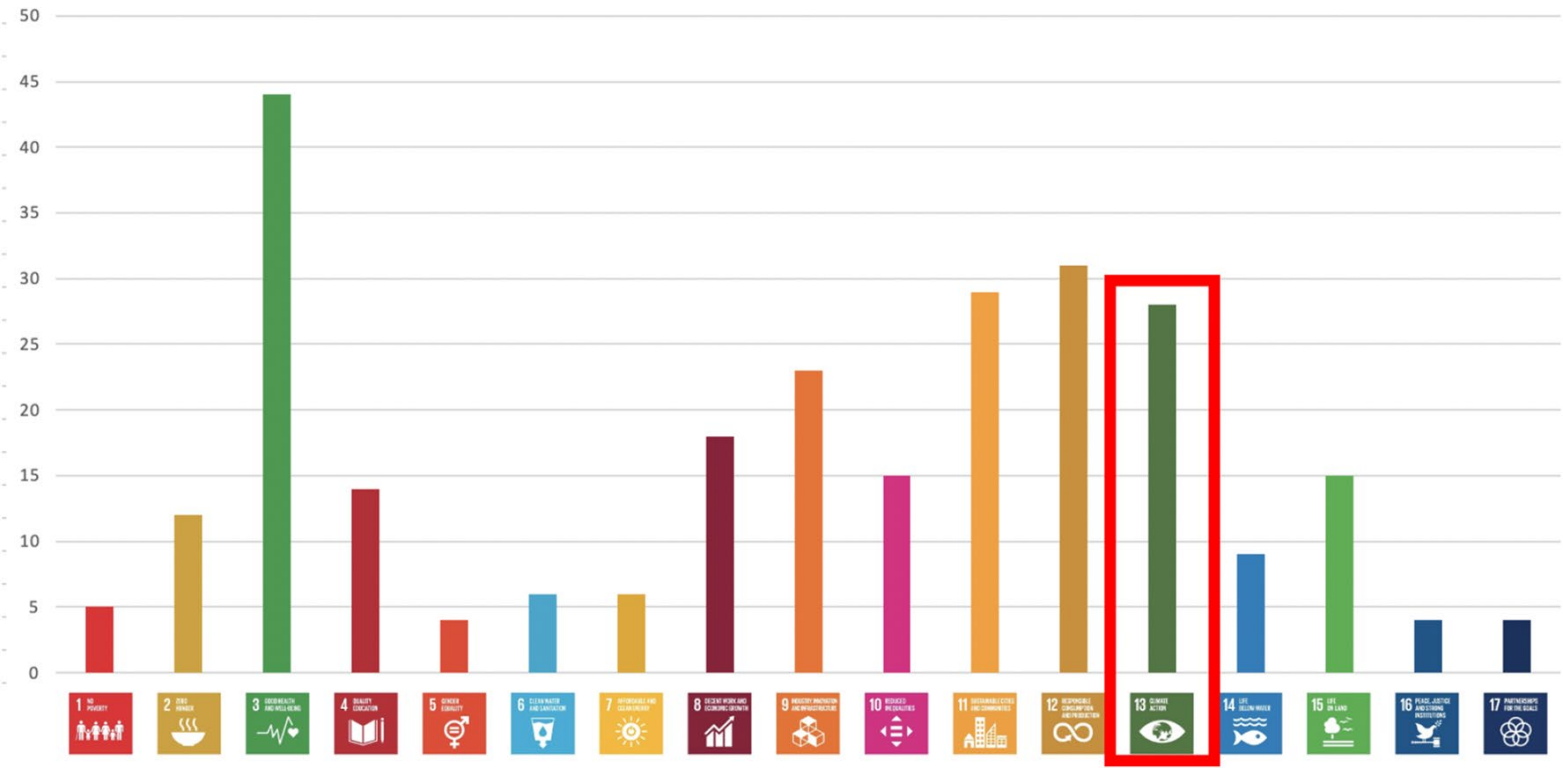
AI-based projects addressing the UN Sustainable Development Goals (Cowls et al. 2021)

Although AI is not a silver bullet nor “the only tool in the drawer” for combating climate change, and while uncritical “solutionism” regarding the use of AI for social good should be avoided (Cowls et al. 2021), nonetheless as the previous section illustrates, efforts to use AI to combat climate change are growing at a fast pace. Because of this pace of development, undertaking a more comprehensive, and rigorous, assessment is a challenge. To date, several systematic approaches to gathering evidence of the use of AI for climate change worldwide have been trialled, resulting in a range of datasets, organised in different ways, each of which paints a partial picture of the phenomenon. For instance, some researchers have used the United Nations Sustainable Development Goals (SDGs) as a basis for evidence-gathering about AI-based solutions to address climate change. Of the 17 SDGs, goal 13, “Climate Action”, is most explicitly associated with climate change, but several others, such as 14, “Life Below Water”, and 15, “Life on Land”, are also related. For example, the database of University of Oxford’s Research Initiative on AIxSDGs^12^ contains 108 projects, of which 28 are labelled as related to Goal 13 (see Fig. 4); the SDG AI Repository managed by the UN’s ITU agency^13^ contains 9 climate-focused projects; and the database of the AI4SDGs Think Tank^14^ contains 5.

Clearly, the full range of projects using AI to tackle climate change around the world is not captured in these databases. This may be a result of the selection criteria employed in the surveys, or a lack of awareness of these evidence-gathering efforts among those actually deploying the technology (despite the annual, high-profile AI for Good summit organised by the ITU). It may also be that the SDGs are not the ideal framework, at least scientifically, for exploring the use of AI to tackle climate change. Each SDG contains specific targets and indicators (five and eight respectively in the case of the 13th goal), which are high-level and policy-focused. Consider, for example, indicator 13.1.2, the “number of countries with national and local disaster risk reduction strategies”. Tying the outputs of a single AI initiative to high-level and policy-focused outcomes may prove to be problematic and make the SDGs less than the ideal framework to map the uses of AI to tackle climate change.

**Fig. 5:**
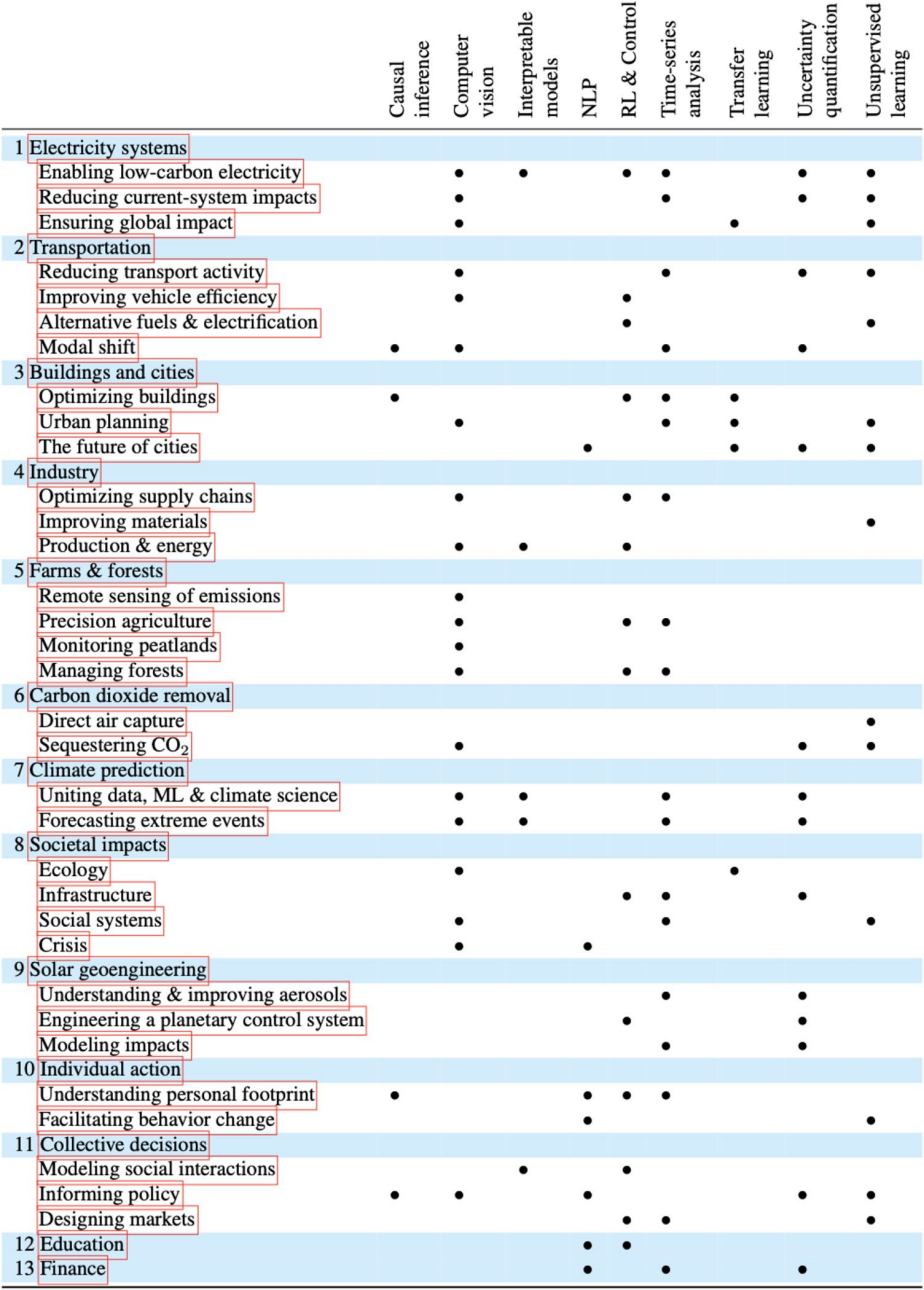
Domains of prospective positive climate impact and forms of AI technology relevant to each, from Rolnick et al. (2019)

Alternative approaches to mapping the uses of AI to address the climate crisis clarify the phenomenon further. One recent large-scale study pinpointed 37 use cases within 13 domains where AI^15^ “can be applied with high impact in the fight against climate change” (Rolnick et al. 2019, 2), and offered a host of examples. For each case, the authors noted which subdomain of the technology (causal inference, computer vision, etc.) could be beneficial (see Fig. 5). Since the publication of this landscaping study, the authors have launched Climate Change AI (CCAI), an organisation composed of “volunteers from academia and industry who believe that tackling climate change requires concerted societal action, in which machine learning can play an impactful role”,^16^ which has resulted in a wide network of researchers.

**Fig. 6:**
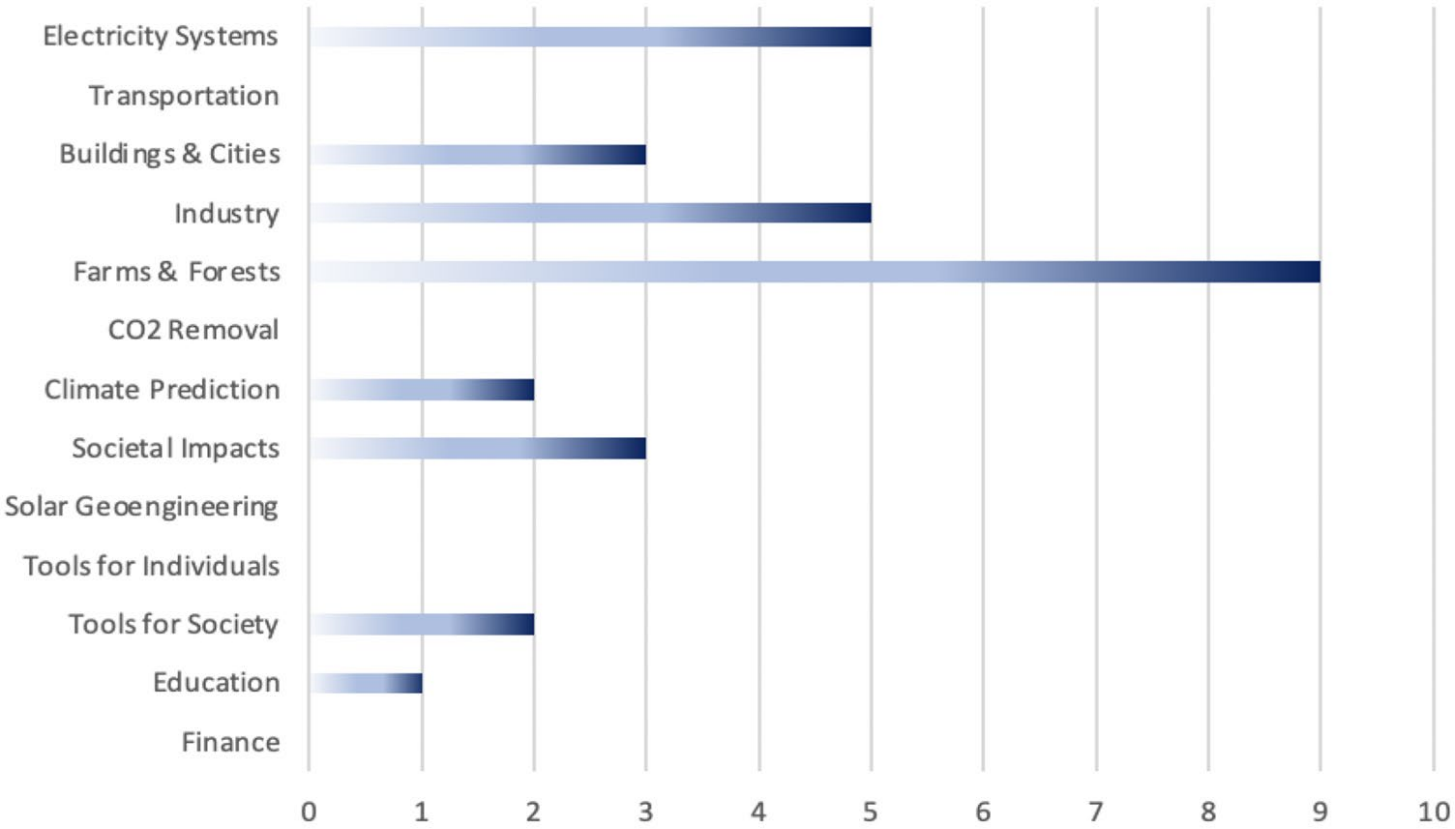
Projects in the Oxford AIxSDG database working in the different domains identified by Rolnick et al. (2019)

Each of these approaches to gathering evidence of AI used to combat climate change helps illuminate the nature of the phenomenon and understand better which domains are attracting more efforts and which are potentially overlooked. Consider for example a cross-analysis (Cowls et al. 2021) between the aforementioned Oxford Research Initiative AIxSDGs database and scoping study by Rolnick et al. (2019). Figure 6 charts the number of climate change-related projects in the AIxSDGs against the specific domains identified by Rolnick and colleagues. In some domains, such as Farms & Forests, there is clear evidence of projects that met the criteria for inclusion in the AIxSDGs database, whereas in others few if any projects are included. This may result in part from the criteria used in the AIxSDGs database collection, among which was the need for evidence of tangible real-world impact.

It is clear that AI offers many options for addressing a wide array of challenges associated with climate change. And given the severity and scope of the challenges posed by climate change, it may be advisable to experiment with a wide array of potential solutions across many domains, as discussed in Sect. 2.1. However, the opportunities offered by AI can only be harnessed to their full potential if ethical values and social expectations are to be met. We turn to these next.

### What are the risks to be avoided or minimised?

Using AI in the context of climate change poses fewer and less severe ethical risks (Tsamados et al. 2020) than using AI in domains such as health and criminal justice, where personal data and direct human-facing decisions are at the core of all activities. Nonetheless, it is important to avoid or minimise the ethical risks that may still arise when maximising the positive impact AI in the fight against climate change.

The first set of risks follows from the way AI models are designed and developed (Yang et al. 2018). Most data-driven approaches to AI are supervised, i.e. they are “trained” on existing labelled data as a basis from which to “learn” to cluster, classify, predict or make decisions regarding new, previously unseen data. This introduces the potential for unwanted bias to enter into the decisions at which an AI system ultimately arrives. This may lead to discrimination and unfair treatment of individuals or groups. Consider, for example, the earlier case of using AI to decide where to locate charging stations for electric vehicles (EVs) based on existing patterns of EV use (Tao et al. 2018). It is possible that using AI to decide where to place charging stations based purely on existing patterns of EV ownership—which could be skewed towards wealthier areas—may result in bias against less wealthy areas, in turn disincentivising the uptake of EVs in these areas. In the same vein, attempts to rely on smartphones to infer individuals’ transportation choices (Dabiri and Heaslip 2018) may lead to biased choices unless communities with lower smartphone uptake are properly accounted for.

A second set of risks concerns the erosion to human autonomy that some climate-focused AI systems may pose (Floridi and Cowls 2019; Taddeo and Floridi 2018). Tackling climate change requires large-scale coordinated action, including systematic changes to individual behaviour. As Rolnick et al. note, “understanding individual behaviour can help signal how it can be nudged” (2019, p. 51), for example through limiting people’s “psychological distance” to the climate crisis, helping them visualise its impacts, or encouraging them to take pro-environmental actions. There is considerable debate over the impact of nudging on individual autonomy (Floridi 2016), and whether it prevents people making “free choices” (for discussion see Schmidt and Engelen 2020), so adopting such an approach in the environmental context requires striking the right balance between protecting individual autonomy and implementing large-scale climate-friendly policies and practices (Coeckelbergh 2020).

Along with fair treatment and autonomy, uses of AI to fight climate change also risk breaching privacy. To the extent to which AI systems rely on non-personal data, e.g. meteorological and geographical data, to understand the climate crisis, they are unlikely to raise privacy concerns. But devising strategies to limit emissions may require data that reveal patterns of human behaviour, where privacy concerns could have more relevance. For example, in control systems designed to decrease carbon footprints in a range of contexts, such as energy storage (Dobbe et al. 2019), industrial heating and cooling (Aftab et al. 2017), and precision agriculture (Liakos et al. 2018), the effectiveness of AI systems depends on granular data about energy demands, often available in real time. The data collected may contain sensitive personal information, risking privacy at both individual and group levels (Floridi 2017). This tension is highlighted in recent Vodafone Institute research finding showing that, while Europeans are broadly willing to share their data to help protect the environment, a clear majority (53%) would only do so under strict conditions of data protection (Vodafone Institute for Society and Communications 2020, 3).

None of these obstacles emerge solely from the use of AI to combat climate change. However, ethical challenges caused by AI may take on novel forms in this context, and, therefore, require careful responses. Furthermore, the computational cost and potential environmental impact of developing AI systems raises a different set of considerations specific to the climate change domain, which are the focus of the next section.

## AI’s carbon footprint

AI (both in the sense of training models and of uses) can consume vast amounts of energy and generate greenhouse gas (GHG) emissions (García-Martín et al. 2019; Cai et al. 2020). This is why establishing systematic and accurate measurements of AI’s carbon footprint is key to ensuring that efforts to harness the potential of this technology outweigh its environmental cost. For reasons explained in Sect. 3.1, this section focuses on methods to estimate the carbon footprint only of AI research (training models), not of AI uses in general, and the technological and normative factors that contribute to the rise of computationally intensive AI research.

Following the advent of deep learning (DL), computing power (henceforth compute) usage rose exponentially, doubling every 3.4 months (Amodei and Hernandez 2018), as specialised hardware to train large AI models became central to the research field (Hooker 2020). The increase in energy consumption associated with training larger models and with the widespread adoption of AI has been in part mitigated by hardware efficiency improvements (Ahmed and Wahed 2020; Wheeldon et al. 2020). However, depending on where and how energy is sourced, stored and delivered, the rise of compute-intensive AI research can have significant, negative environmental effects (Lacoste et al. 2019).

### Gauging the carbon footprint of AI

A “carbon footprint” accounts for the GHG emissions of a device or activity, expressed as carbon dioxide equivalent (CO_2_eq). When applied to a product like a smartphone, a carbon footprint estimation considers emissions that occur during constituent activities, like the extraction of raw materials, manufacturing, transportation, lifetime usage and how the product is disposed of (Crawford and Joler 2018; Malmodin and Lundén 2018). This estimate includes, among other things, information on the carbon/emission intensity of electricity generation throughout a product’s lifecycle and on the carbon offsetting efforts made by the various actors involved in the aforementioned activities (Matthews et al. 2008). However, determining the carbon footprint of a type of product (e.g. AI systems) or entire sector (e.g. Information Communication Technologies, ICT) can be a daunting task that yields only partial results, not least due to transparency issues and methodological challenges of GHG monitoring (Matthews et al. 2008; Russell 2019; Cook and Jardim 2019; Mytton 2020).

Estimates of GHG emissions of the ICT sector (including computing devices and data centres) vary greatly across different studies (Malmodin and Lundén 2018; Hintemann and Hinterholzer 2020). Malmodin and Lundén’s (2018), a widely cited study based on data from 2015, estimates that the ICT sector is responsible for 1.4% of global GHG emissions. Depending on future efficiency gains and the diversification of energy sources, estimates indicate that the ICT sector will be responsible for anywhere between 1.4% (assuming a stagnant growth) to 23% of global emissions by 2030 (Andrae and Edler 2015; Malmodin and Lundén 2018; C2E2 2018; Belkhir and Elmeligi 2018; Jones 2018).^17^ At the same time, it is worth noting that the demand for data centres, which are key to the ICT sector and the operation of AI in research and production settings, has grown substantially in recent years, yet data centres’ energy consumption has remained relatively stable (Avgerinou et al. 2017; Shehabi et al. 2018; Jones 2018; Masanet et al. 2020). The International Energy Agency reports that, if current efficiency trends in hardware and data centre infrastructure can be maintained, global data centre energy demand—currently 1% of global electricity demand—“can remain nearly flat through 2022, despite a 60% increase in service demand” (IEA 2020). Indeed, significant efforts have been made to curb data centres’ carbon footprint by investing in energy-efficient infrastructure and switching to renewable sources of energy (Jones 2018; Masanet et al. 2020). Cloud providers especially, such as Microsoft Azure and Google Cloud, have worked to keep their carbon footprint in check by committing to renewable energy, improving cooling systems and efficient processors, recycling waste heat, and investing in carbon offsetting schemes (Jouhara and Meskimmon 2014; Avgerinou et al. 2017; Jones 2018; Open Compute Project 2020). In fact, both providers have leveraged AI to reduce the energy consumption of their data centres, in some cases by up to 40% (Evans and Gao 2016; Microsoft, C 2018).

Whether these efforts keep pace with the growing demand for data centre services and whether efficiency gains are equally realised around the world will be crucial factors affecting the environmental impact of the sector. These goals may not be easily achievable. Even in the EU, where energy-efficient cloud computing has become a primary issue on the political agenda, the European Commission estimates a 28% increase in energy consumption of data centres by 2030 (European Commission 2020d). Things are complicated even further by transparency concerns regarding the data required to calculate GHG emissions of on-premise data centres as well as cloud vendors, which will need to be addressed to obtain an accurate understanding of the carbon footprint of the ICT sector (Hintemann 2015; Mytton 2020; Hintemann and Hinterholzer 2020).

At the same time, understanding the carbon footprint of AI involves more than just monitoring data centres, as the rest of this section will show (Henderson et al. 2020; Cai et al. 2020). Given the wide range of artefacts and activities relying on some form of AI and the multi-layered production process of AI systems—spanning from data collection and storage, to hardware production and shipment, to AI/machine learning (ML) model trainings and inferences—gauging the carbon footprint of AI is challenging. This is why this section focuses on the carbon footprint associated with the energy consumption of AI research activities, available in corresponding research publications.^18^ As we shall see in Sect. 3.3, verifiable information on the short- and projected medium-term environmental impact of AI research activities is limited and suffers from a lack of systematic and accurate measurements. However, the information contained in research publications regarding the energy consumption and carbon emission of AI is more accessible and testable than in industry reports. Thus, it offers a more reliable starting point to understand the environmental impact of AI, even if it is indicative only of a subset of all AI-related activities. Furthermore, to gauge the energy consumption and carbon footprint of AI research activities, it is important to distinguish between two phases of computation that are central to supervised ML research methods: training (or “learning”) and inferences. Training a ML model involves providing labelled sample data, or a “training set”, to a ML algorithm so that it can “learn” from it and create an appropriate mathematical model with the optimal parameters that minimise a certain cost function (e.g. some metric of error). Once the training phase is finished, a model and its parameters are fixed and such model can be operationalised and produce actionable output on new, unseen data, which is the “inference” process.

In the short term, the training phase is computationally more demanding and energy intensive (Al-Jarrah et al. 2015). In the medium term, the energy consumption of the inference phase scales with usage, as inference can usually occur millions of times per day for an indefinite amount of time (Sze et al. 2017). So, training is often more energy-intensive in data-driven, ML-based research, while inference might be more energy-intensive in at-scale production systems which may require non-stop usage. This is why, in the context of AI as a whole, this article focuses on information pertaining to the research and training of AI models.

Several approaches to monitoring and estimating the GHG emissions of AI research activities have been recently offered. These include the reporting of floating point operations (Lacoste et al. 2019; Schwartz et al. 2019; Henderson et al. 2020), hardware type and hardware burden or “processors multiplied by the computation rate and time” (Thompson et al. 2020, 10), the data centre in use during model training, as well as the energy sources powering the electrical grid (Schwartz et al. 2019; Anthony et al. 2020), the number of experiments required during model construction (Schwartz et al. 2019; Strubell et al. 2019), and the time period in which a model was trained, as carbon/emission intensity can vary throughout the day (Anthony et al. 2020)*.* Of these approaches, two recent efforts stand out for their generalisability and/or ease of use, namely Henderson et al.’s (2020) *“*experiment-impact-tracker” and Lacoste et al’s (2019) Machine Learning Emissions Calculator.

The first approach rests on a comprehensive framework available on GitHub (Henderson et al. 2020), specifying the relevant data to collect during and after model training phases to assess the related GHG emissions:

1. Central processing unit (CPU) and graphics processing unit (GPU)^19^ hardware information;
2. experiment start and end times;
3. the energy grid region the experiment is being run in (based on IP address);
4. the average carbon/emission intensity in the energy grid region;
5. CPU- and GPU-package power draw;
6. per-process utilisation of CPUs and GPUs;
7. GPU performance states;
8. memory usage;
9. the real-time CPU frequency (in Hz);
10. real-time carbon intensity.
11. disk write speed.

Unfortunately, information about these 11 variables is rarely available in its entirety in most research publications (Henderson et al. 2020). In an analysis of 1,058 research papers on DL, Thompson et al. (2020, 10) found that most papers “did not report any details of their computational requirements”.

By contrast, the second approach (Lacoste et al.’s 2019) limits itself to information pertaining to the type of hardware, hours of training, region of compute, and cloud provider/private infrastructure. This is a helpful approach to estimating the carbon footprint of AI research activities using a minimum amount of data and without actually reproducing experiments and models. For this reason, we use Lacoste et al.’s (2019) approach to calculate the carbon footprint of large AI research projects, and we use GPT-3, OpenAI’s latest research breakthrough as our case study. While the following estimates cannot be definitive, due to a lack of data available in OpenAI’s research publication, they serve to reflect both the importance and the difficulty of assessing carbon footprints when researchers fail either to report them or to provide enough information regarding training infrastructure and model implementation.

GPT-3 is an autoregressive language model that has attracted considerable attention from researchers and news outlets since documentation was published on arXiv in May 2020 by Brown et al. (2020). From the research publication detailing GPT-3, we know that the model required several thousands of petaflop/s-days (3.14E23 FLOPS) of compute during pre-training. This is orders of magnitude higher than the previous state-of-the-art (SOTA) 1.5B parameter GPT-2 model that the company released in 2018, which required only *tens* of petaflop/s-days (Radford et al. 2018). GPT-3 was trained using NVIDIA’s V100 GPUs on a cluster provided by Microsoft. Thus, one can calculate that, at a theoretical processing speed of 28 Terra Flops (TFLOPS)^20^ for a V100 GPU, it would take around 355 GPU years for a single training run (Li 2020).

**Fig. 7:**
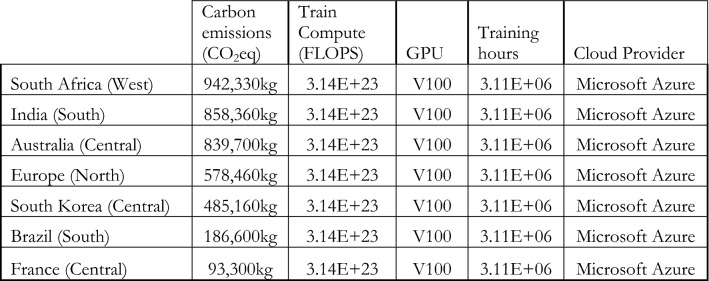
Environmental costs (in kg of CO_2_eq) of a single training run of GPT-3 across different compute regions (Regional carbon intensity sourced from https://github.com/mlco2/impact/tree/master/data.)

Using Lacoste et al.’s carbon impact calculator and assuming that the cloud provider (Microsoft Azure) was based in the US (West), we find that a single training run would have generated 223,920 kg CO_2_eq. If the cloud provider had been Amazon Web Services (AWS), the same training would have generated 279,900 kg CO_2_eq.^21^ This does not include the carbon offsetting efforts made by these companies (Mytton 2020). As a point of reference, a typical passenger car in the United States emits about 4600 kg CO_2_eq per year (US EPA 2016), meaning that one training run would emit as much as 49 cars (Microsoft Azure) or 61 cars (AWS) in a year. A single training run can emit drastically more GHG depending on the region of compute and the carbon/emission intensity of electricity generation in the selected region (Lacoste et al. 2019). For example, it is ten times more costly in terms of CO_2_eq emissions to train a model using energy grids in South Africa compared to France (see compute regions in Lacoste et al. 2019). Figure 7 below offers examples of the variation of energy consumption across different countries.

The authors of GPT-3 (Brown et al. 2020) also note that training the model required an immense amount of resource, but GPT-3 has the advantage of adapting to new tasks quite efficiently compared to other language models that would be relatively costly to fine-tune. For example, the authors report that to generate “100 pages of content from a trained model can cost on the order of 0.4 kW-h, or only a few cents in energy costs” (Brown et al. 2020).

More recently, researchers from the Google Brain team released a research paper stating to have trained a 1.6 trillion parameters language model—approximately 9 times bigger than GPT-3 (Fedus et al. 2021). And although the paper describes the use of a training technique that reduces computational costs and increases model training speed, it does not indicate the energy consumption or carbon emissions of the research project. This comes against the backdrop of earlier warnings from Google’s own Ethical AI team regarding the environmental costs of such large models (Bender et al. 2021).

It is crucial for the field of AI to come to terms with these numbers. These large AI research projects may be indicative of—and exacerbate—a failure to engage with environmental questions, to disclose important research data, and to shift the focus away from ecologically short-sighted success metrics (García-Martín et al. 2019; Schwartz et al. 2019; Henderson et al. 2020). In what follows we explore the technological and normative factors that have entrenched the field of AI research on an energy-intensive, and potentially carbon-intensive, path.

### Factors driving increases in AI’s carbon footprint

#### Technological considerations: compute-intensive progress

The recent rise of AI can be largely attributed to the increasing availability of massive amounts of data and to the adoption of general methods leveraging the “continued exponentially falling cost per unit of computation” described by Moore’s law (i.e. the number of transistors per microchip doubles every 2 years for the same costs) (Sutton 2019). DL epitomises AI research that is based on scaling general purpose methods with increased computation and availability of large amounts of unstructured data (Sutton 2019). Recent breakthroughs, where AI models were able to reach parity with humans on a number of specific tasks, are the result of such AI research based on deep neural networks and improvements in computation and data availability (Ahmed and Wahed 2020; Hooker 2020). However, the advent of DL has also marked a split between the increase in available compute (i.e. Moore’s law) and the increase in compute*-*usage (Theis and Wong 2017; Thompson and Spanuth 2018; Ahmed and Wahed 2020). Exploring these trends helps us map the risks and opportunities of AI research with regards to climate change.

**Fig. 8:**
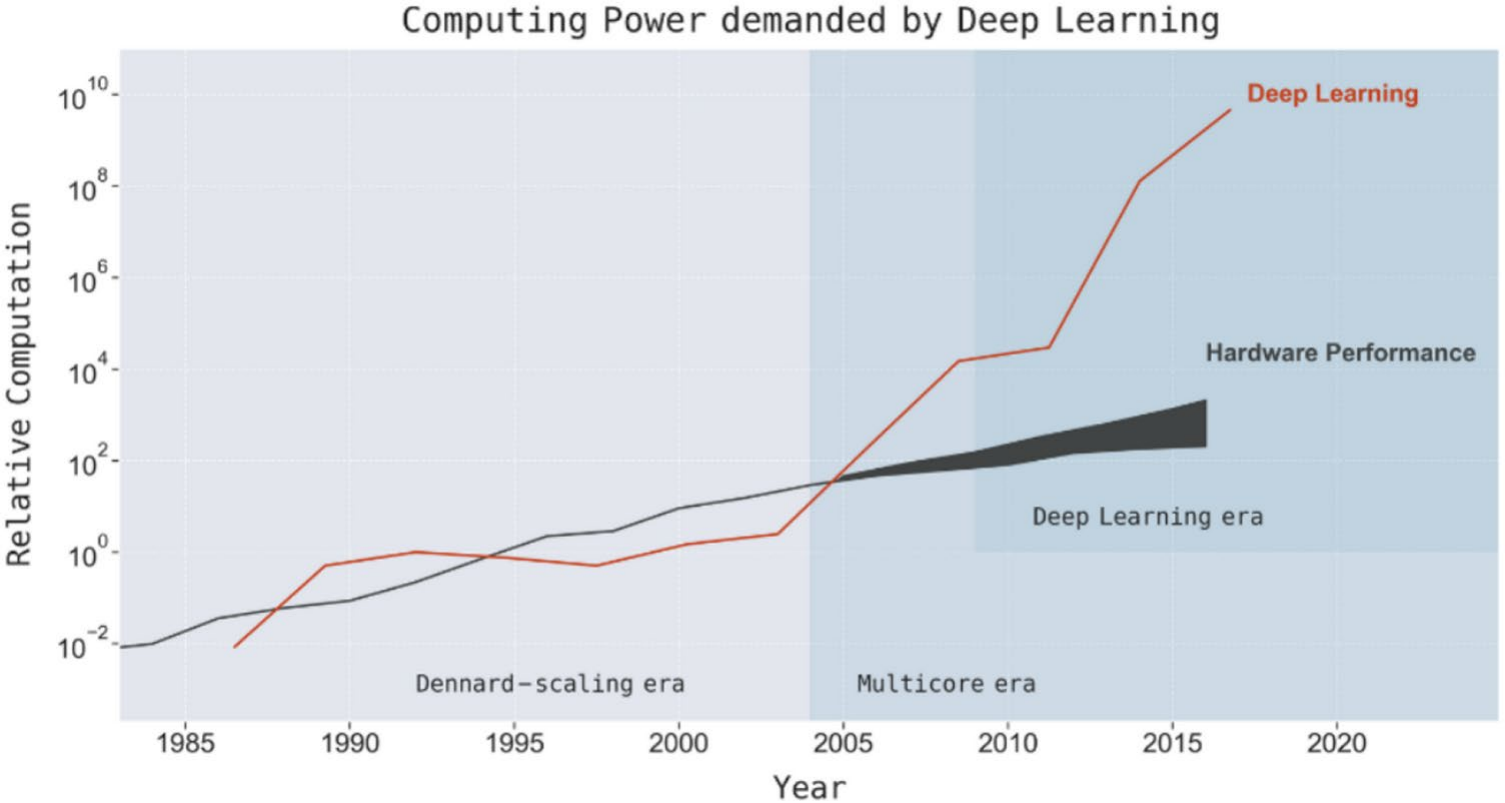
Computing power demanded by DL throughout the years (figure taken from Thompson et al. 2020)

Moore’s law has resulted in developers being able to double an application’s performance for the same hardware cost. Prior to 2012, AI developments have closely mirrored Moore’s law, with available compute doubling approximately every two years (Perrault et al. 2019). As shown in Fig. 8, improvements in computer hardware provided almost a 50,000 × improvement in performance, while the computational requirements of neural networks had grown at a similar pace until the introduction of chips with multiple processor cores (Hill and Marty 2008; Thompson et al. 2020, 8). Arguably, hardware development has often determined what research activities would be successful (Hooker 2020). For example, deep convolutional neural networks and backpropagation, which are central components to contemporary DL research, had already been introduced in the 80s (Fukushima and Miyake 1982; Werbos 1988), but had real impact only four decades later, following hardware progress and large-scale data availability (Hooker 2020).

As described by Thompson et al. (2020, 8), during the “multicore era”, DL was “ported to GPUs, initially yielding a 5 − 15 × speed-up which by 2012 had grown to more than 35 × ” and which led to the AlexNet breakthrough in 2012 (Alom et al. 2018). Shortly after the AlexNet breakthrough in image recognition, a number of achievements followed in the various subfields of AI. In 2015, a reinforcement learning (RL) system achieved human-level performance in a majority of Atari games; in 2016 object recognition reached human parity and AlphaGo beat one of the world’s greatest Go players; in 2017 speech recognition reached human parity; in 2018 reading comprehension, speech synthesis and machine translation all reached human parity; and in 2019, the ability to scan and extract contextual meaning from text and speech (and answer a series of interconnected questions) reached human parity (Alom et al. 2018; Microsoft 2019; Evans and Gao 2016). These breakthroughs were all possible due to considerable increases in compute-usage (Ahmed and Wahed 2020). Indeed, since 2012, compute-usage has been doubling every 3.4 months, spearheaded by the development of DL (Amodei and Hernandez 2018).

Increases in compute have been essential, especially to RL, as this is an area of ML that stands out for its sample-inefficient methods of learning. Learning phases can require hundreds of millions of samples, making it impractical for “real-world control problems” such as in robotics (Buckman et al. 2018). Yet, RL has been used for text summarisation, robotic manipulation, and also to compete with human performance in domains such as Atari games, Chess, and Go (Berner et al. 2019). As researchers begin to apply RL methods to increasingly complex domains, like online multiplayer games, sample inefficiency will continue to drive energy costs higher. For example, OpenAI Five, which was developed to compete with professional Dota 2 players, played 900 years’ worth of games per day to reach a competitive level at the game (Berner et al. 2019). After ten months of training, using around 770 Peta Flops/s·days of compute, the model beat the world champions at Dota 2 (Berner et al. 2019).

**Fig. 9:**
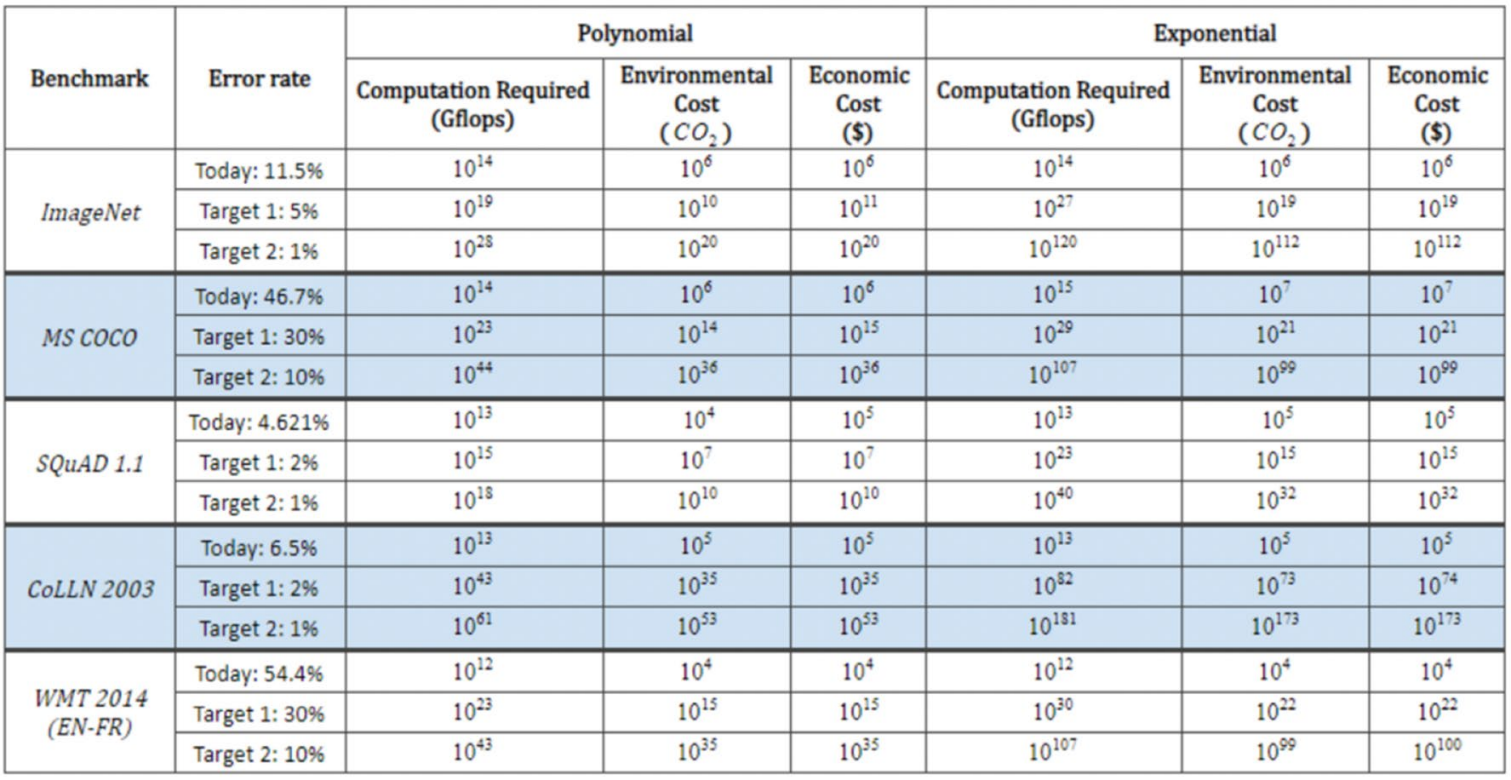
Implications of achieving performance benchmarks on the computation, carbon emissions (lbs), and economic costs from deep learning based on projections from polynomial and exponential models (figure from Thompson et al. 2020)

The multicore era also marks a decoupling of the improvements in hardware performance from the growth in computational requirements of large AI models, with the latter considerably outpacing the former. Because of this, researchers are facing diminishing returns (Thompson et al. 2020). The compute needed to train SOTA models is growing approximately ten times faster than GPU performance per watt (Thompson et al. 2020). This means that the present trend in scaling ML models is unlikely to be a sustainable path forward, both in terms of financial costs and for the preservation of the planet, given the very high levels of energy consumption that are associated with it (Henderson et al. 2020; Thompson et al. 2020). As shown in Fig. 9, it would be financially and ecologically prohibitive to reach lower error rates in different tasks, as any improvement (measured in percentage points) on a model’s accuracy would require significantly more energy and GHG. For example, if we look at the Thompson et al.’s (2020) polynomial models, it appears that reducing the error rate by 16.7 percentage points for MS COCO (Common Objects in Context) to achieve an error rate of 30%, would require 10^9^ × more computation (GFLOPS) and generate 10^8^ × more CO_2_eq (in lbs).

To enable the current compute-usage trend and mitigate diminishing returns, ML-specific hardware, such as Google’s TPUs, and various approaches, like neural architecture search, have been developed in recent years (Amodei and Hernandez 2018). However, even as new devices and hardware architectures continue to deliver better energy efficiency, it is not guaranteed that these improvements will keep pace with compute-usage, nor are they guaranteed to be available to everyone around the globe (Ahmed and Wahed 2020; Hooker 2020). It follows that, if AI researchers are unable to access SOTA hardware to train large ML models, or if hardware performance does not keep pace with the growth of compute-usage in AI research, then the field’s energy consumption will grow quickly (Nature Electronics 2018). Additionally, research has shown that the current focus on DL and custom hardware has come at the detriment of funding “hardware for use cases that are not immediately commercially viable”, making it more costly to diversify research (Hooker 2020, 9).

Algorithmic progress has also shown promising effects in relation to efficiency improvements for large model trainings. Although algorithmic progress is more dependent on human knowledge—as opposed to computational advances—and thus takes more time and effort to occur (Sutton 2019), Thompson et al. (2020) note that three years of algorithmic improvement is equivalent to an increase in computing power by a factor of 10. This can be observed in image recognition (Hernandez and Brown 2020), neural machine translation (Thompson et al. 2020), and certain areas of RL (Hernandez and Brown 2020). For example, since 2012, the compute required to train a neural network to the “same performance on ImageNet classification has been decreasing by a factor of 2 every 16 months” and it now takes “44 times less compute to train a neural network to the level of AlexNet” (Hernandez and Brown 2020). Nevertheless, we note that research exploring new neural network architectures or new hardware–software–algorithm combinations has largely been side-lined in favour of compute-intensive AI research (Hooker 2020; Marcus 2020; Ahmed and Wahed 2020).

Fortunately, researchers have also sought to reduce the computational burden and energy consumption of AI by focusing on building more efficient models through various approaches, such as random hyperparameter search, pruning, transfer learning or simply by stopping training early for underperforming models (Sze et al. 2017; Pham et al. 2018; Chen et al. 2019; Schwartz et al. 2019; Coleman et al. 2019a). More efforts are required in these areas. To be successful they need endorsement and cultivation from the wider field of AI to gain larger uptake. For AI research to continue to thrive, while keeping its carbon footprint in check and avoid running into a technological impasse in the coming years (Jones 2018), the field needs to reconsider its dedication to compute-intensive research and move away from performance metrics that focus exclusively on accuracy improvements (Schwartz et al. 2019). The following section addresses the normative factors that have enabled these negative trends.

#### Normative considerations

For a field of research that relies on data collection and data processing, information about the energy consumption and carbon emissions of AI/ML models and research activities should be more detailed and more accessible (Henderson et al. 2020). Indeed, alongside the technological factors that have skewed the development of AI/ML models, there are also normative factors, like the lack of effective reproducibility requirements for research publications, which also contribute to explain the entrenchment of research activities in energy-intensive practices (Hooker 2020; Fursin 2020). Examining these factors, and questioning the validity of some standards and practices in AI research, is key to ensuring that the field keeps its carbon footprint to a minimum.

AI research has been grappling with a reproducibility crisis. Given the growing amount of AI-related research activities and compute-usage, this crisis needlessly supercharges the field’s carbon footprint (Fursin 2020). From papers that do not disclose their code (as is the case for GPT-3) to papers that do not share the data used to train their model (e.g. for privacy or proprietary reasons) to papers that provide insufficient or even misleading information about the training conditions of their models, there have been persistent obstacles to verifying and reproducing results in AI research (Gibney 2020; Fursin 2020). In turn, these obstacles translate into unnecessary energy consumption.

After conducting a survey of 400 algorithms presented in research papers at two top AI conferences (IJCAI and NeurIPS), researchers reported that only 6% of the presented papers shared the algorithm's code, a third shared the data on which they tested their algorithms, and only half shared a partial summary of the algorithm (Gundersen and Kjensmo 2018; Hutson 2018). Several studies have investigated this issue in the context of energy consumption and carbon emissions (Lacoste et al. 2019; Schwartz et al. 2019; Strubell et al. 2019; Henderson et al. 2020; Dhar 2020). Indeed, after analysing a sample of 100 papers from the NeurIPS 2019 proceedings, Henderson et al. (2020, 4) reported that none of them provided carbon metrics, only one of them “measured energy in some way, 45 measured runtimes in some way, 46 provided the hardware used” and 17 of them “provided some measure of computational complexity (e.g., compute-time, FPOs, parameters)”. Although major AI conferences, such as ICML, IJCAI or NeurIPS, are increasing their efforts to normalise the submission of code and have implemented reproducibility checklists, the disclosure of information regarding computational complexity, energy consumption, and carbon emission is still uncommon (Strubell et al. 2019; Thompson et al. 2020).

Sharing source code is necessary to ensure reproducibility in AI research. But it is not sufficient. Researchers have highlighted the importance of disclosing the training data and the initial parameters set for the training phase, or hyperparameters (Schwartz et al. 2019; Hartley and Olsson 2020). Sharing a model’s sensitivity to hyperparameters, or of the random numbers generated to start the training process in the case of RL, is essential to allow researchers to reproduce results without going through a long, and environmentally costly, process of trial and error (Hutson 2018; Strubell et al. 2019; Gibney 2020). Indeed, the number of experiments run by researchers before achieving publishable results are both “underreported and underdiscussed” (Dodge et al. 2019; Schwartz et al. 2019, 9). In this case, a direct result of incomplete or misleading information disclosures is the “double costs” incurred by researchers that have to rediscover, even if only partially, the information that led to the reported results. Building on existing research becomes more difficult when newcomers have to incur unnecessary costs of experimentation that were already incurred for the original publication of a model. This approach inflicts an unnecessary double cost on the environment via increased energy consumption.

According to recent research on the energy consumption and carbon footprint of DL in natural language processing (NLP), the process of researching and developing SOTA models multiplies the financial and environmental costs of training a model by thousands of times (Strubell et al. 2019). Indeed, over the course of six months of research and development, a single research paper may require training thousands of models before being published (Dodge et al. 2019; Schwartz et al. 2019, 4). Similarly, Schwartz et al’ (2019, 4) have reported that massive amounts of computation go into “tuning hyperparameters or searching over neural architectures”. This is the case, for example, of Google Brain, which trained over 12,800 neural networks in its neural architecture search to achieve a 0.09 percent accuracy improvement and 1.05 × in speed on the CIFAR-10 dataset (Zoph and Quoc 2017). In light of our calculations regarding the carbon emission of a single training run for GPT-3, this would mean that to achieve their published model the research team at OpenAI may have generated much more CO_2_eq than previously estimated. Failing to report the research experiments that went into achieving the reported results can have a snowball effect for the field of AI research in terms of energy consumption and carbon emissions, as it imposes a longer trial-and-error process onto new researchers.

Modern AI research has focused on producing deeper and more accurate models at the detriment of energy efficiency (Sutton 2019; Perrault et al. 2019; Hooker 2020). Indeed, some of the main benchmarks, challenges and leader boards on which AI researchers and organisations compete, such as GLUE (2020), SuperGLUE (2020), SQuAD2.0 (2020), Russakovsky (2015) and VTAB (2020), have been heavily focused on driving accuracy improvements with little regard for improving on energy efficiency (Perrault et al. 2019; Reddi et al. 2020). This narrow focus increases compute-intensive AI research and exacerbates diminishing returns, with researchers competing for fractional improvements in error rates (Henderson et al. 2020). It is only relatively recently that efforts have emerged to reduce compute-usage and improve energy efficiency of DL methods, at the algorithmic, hardware, as well as implementation levels (Chen et al. 2015, 2017; EDL 2017; Sze et al. 2017; Guss et al. 2019; García-Martín et al. 2019; Jiang et al. 2019; Cai et al. 2020). The Low Power Image Recognition Challenge (LPIRC) is a good example of such efforts (García-Martín et al. 2019).

**Fig. 10:**
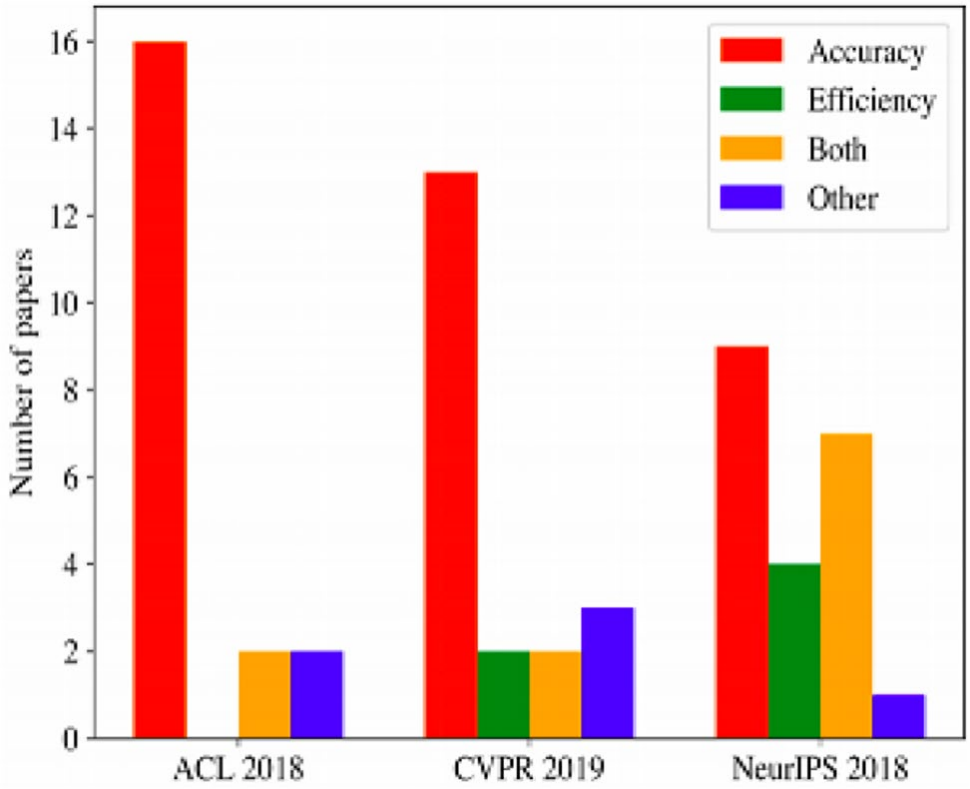
Proportion of papers that target accuracy, efficiency, both or other from a sample of 60 papers (figure from Schwartz et al. 2019)

To demonstrate the prevalence of accuracy metrics over efficiency metrics, a group of researchers at the Allen AI Institute sampled 60 papers from top AI conferences (ACL, CVPR and NeurIPS) that claimed to achieve some kind of improvement in AI (Schwartz et al. 2019). As shown in Fig. 10, a large majority of the papers target accuracy (90% of ACL papers, 80% of NeurIPS papers and 75% of CVPR papers), and in both ACL and CVPR, which are empirical AI conferences, only 10% and 20% respectively argue for new efficiency results (Schwartz et al. 2019). The prevalence of accuracy over efficiency in AI research has also been stressed by the Electronic Frontier Foundation’s “AI Progress Measurement” project, which tracks progress on problems and metrics/datasets from the AI research literature and provides a comprehensive view of the field’s priorities (EFF 2017).^22^

**Fig. 11:**
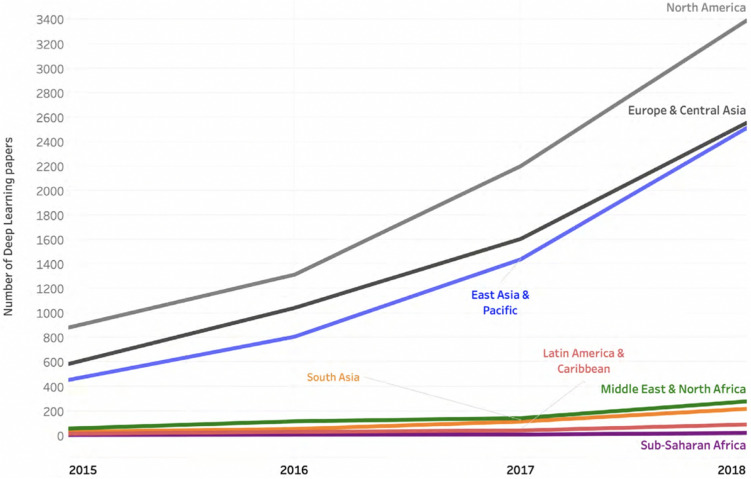
Number of deep learning papers on arXiv, per region (figure from Perrault et al. 2019)

Several issues arise from focusing on accuracy over efficiency metrics. First, it creates a high barrier to entry, as only wealthy research groups are able to incur in the growing costs of compute-intensive research (Ahmed and Wahed 2020). This leads to a limited number of researchers to be able to afford stronger results and hence publications (Schwartz et al. 2019, 2), thus creating a virtual monopoly on fundamental research and side-lining researchers from smaller organisations, less funded contexts, and developing countries (see Fig. 11). Second, it ingrains a “the bigger the better” mentality into the field, thus giving carte blanche to organisations and researchers to accelerate experimentation and increase their eventual energy consumption. This, in turn, makes it harder to explore efficiency improvements. It also reduces the diversity of research topics. Third, it keeps the field on a path of diminishing returns and incentivises researchers to pursue incremental improvements and “publish at all cost”, even if it means achieving practically (for deployed systems) negligible accuracy improvements.

### Overall balance

It is important to keep in mind that, although training AI/ML models can require a lot of energy, they are usually used to improve the efficiency of many tasks that would otherwise require more time, space, human effort, and potentially electricity (Narciso and Martins 2020). When deployed in production settings or edge devices, AI systems can have downstream effects that counterbalance their own energy consumption and GHG emissions (see Sect. 2). Additionally, recent progress in making the deployment of deep neural networks on edge devices, like smartphones and tablets, much more efficient, has been significant for the environmental impact of AI (Cai et al. 2020). Indeed, the diversity of hardware platforms in use today has created various efficiency constraints requiring, for example, that neural networks are redesigned and retrained for each new environment they are deployed in (Cai et al. 2020). However, novel approaches, such as “Once-for-All” networks, show great promise as they require approximately 1/1300 of the carbon emissions of SOTA neural architecture search approaches while also reducing inference time (Cai et al. 2020).^23^

For most or all industry sectors, AI offers significant “gains in efficiency and performance” (Hall and Pesenti 2017, 2), and indeed, the European Commission’s Horizon 2020 programme has been investing in projects using AI systems to improve the energy and resource efficiency of many sectors (Dahlquist 2020). Balancing the energy consumption of AI against its energy-efficiency gains will be an important task for both researchers and regulators alike, one that begins with obtaining enough information about a model.

Our analysis thus far is testament to the complexity inherent to any attempt to talk about AI in the context of climate change. On the one hand, the power of AI can be harnessed to address some of the most complex tasks associated with combating climate change successfully. On the other hand, the development of AI is itself contributing to GHG emissions that advance climate change still further. Taken together, this analysis suggests the need for coordinated, multilevel policymaking that can advance the use of AI to combat climate change, whilst ensuring that the development of AI does not itself contribute to the existing problem. This is why in the remainder of article we turn from technical to political considerations for AI and climate change.

## Policy context: the EU’s twin transitions

The opportunities presented by AI for tackling climate change are just one example of the broader intersection between the digital revolution and the efforts for sustainability. In recent years, this “Green & Blue” formula (Floridi and Nobre 2020; Floridi 2020) has become apparent, at least on paper, in European policymaking. “A European Green Deal” and “A Europe fit for the digital age” were two of the six “headline ambitions” highlighted in the political guidelines released as part of von der Leyen’s campaign. Since von der Leyen took office, documents issued by the Commission have begun referring to the “twin transitions”—ecological and digital—that will shape Europe’s medium- to long-term future. The proximity and interdependence of “green plus blue” has recently been further reinforced by the Commission’s response to the coronavirus pandemic, which anticipates a large stimulus including “modernisation” through “fair climate and digital transitions, via the Just Transition Fund and the Digital Europe Programme” (European Commission 2020i). References to the role of digital technology are studded throughout the “European Green Deal” communication and roadmap, released in December 2019 (European Commission 2019). As the document notes, “digital technologies are a critical enabler for attaining the sustainability goals of the Green Deal in many different sectors”, and technologies “such as artificial intelligence … can accelerate and maximise the impact of policies to deal with climate change and protect the environment” (p. 9). Domains in which “smart” or “innovative” digital technologies are expected to play a role include energy grids (p. 6), consumer products (p. 8), pollution monitoring (p. 9), mobility (p. 10), and food and agriculture (p. 12)—that is, many of the domains in which the existing evidence, summarised in Sect. 2, suggests that AI is already being deployed and will make an increasing difference.

Recent Commission documents on Europe’s forthcoming “digital transformation” equally highlight the possibilities this transformation holds for sustainability. As the Commission’s recently released “Strategy on shaping Europe’s digital future” (European Commission 2020e) notes, digital technologies will “be key in reaching the ambitions of the European Green Deal and the Sustainable Development Goals” as “powerful enablers for the sustainability transition” (European Commission 2020c, p. 5). The document highlights sectors including agriculture, transport and energy as benefiting particularly from digital “solutions”. In addition, other Commission documents released within the last twelve months also highlight the twin transitions:

- The “European strategy for data” announces the establishment of a “common European Green Deal data space” to use shared data to meet Green Deal targets (European Commission 2020b);
- The “White Paper on Artificial Intelligence: A European approach to excellence and trust” highlights the impact of AI on climate change mitigation and adaptation in its first paragraph and again throughout (European Commission 2020g); and
- The “New industrial strategy for Europe” asserts that Europe “needs an industrial sector] that becomes greener and more digital” (European Commission 2020b, p. 2)

Several of the documents make reference to the so-called “Destination Earth” initiative, the stated intention of which is to “develop a very high precision digital model of the Earth to monitor and simulate natural and human activity, and to develop and test scenarios that would enable more sustainable development and support European environmental policies” (European Commission 2020h). Destination Earth is designed to contribute both to the Commission’s Green Deal and to its Digital Strategy. It targets national authorities to aid policymakers and then opens up to users from academia and industry. The technical details of Destination Earth remain to be specified, but it is said to provide access to “data, advanced computing infrastructure, software, AI applications and analytics”. Therefore, while the exact role of AI tools within the initiative remains to be seen, the scale and ambition of Destination Earth and its role at the intersection of the “twin transitions” suggest it may be important in fostering the use AI to tackle climate change.

Given the prominence of digital technologies in everyday life and the increasing salience of the climate change challenge—as well as the coordination of policy priorities that accompanies a new administration—it is not surprising to find concordance among these documents. Even so, the extent to which the Commission seems to anticipate the twin transitions developing hand-in-hand is striking. The EU’s renewed commitment to using AI and other digital technologies to make European society and industry greener and more sustainable is an important statement of intent and suggests that Europe may become a focal point of efforts to develop AI to combat climate change effectively. However, it is important not to conflate the stated aspirations of policymakers with the actual outcomes of policies, especially in the fast-changing and unpredictable domains of digital technology and global climate change. And it could just as well be the case that, despite high hopes for complementarity and coherence between the EU’s digital and ecological agendas, incongruity and conflict may be just as likely to result. The policy documents also tend to presume a harmonious relationship between the digital and ecological transitions, overlooking the trade-offs that may need to be struck between them, and how this could or should be done. For all the opportunities these policy documents highlight, they brush over the challenges that need to be addressed to ensure a successful adoption of AI tools. To this end, in the next section, we offer 13 recommendations.

## Recommendations for EU policymakers and the AI research community

The previous sections identified two areas where recommendations for leveraging the opportunities and addressing the challenges posed by AI in the context of climate change can be offered. Stated as objectives, these are, first, to harness the potential of AI for understanding and combatting climate change in ways that are ethically sound; and second, to gauge and minimise the size of AI’s carbon footprint. In this section, we address these two objectives, to identify specific methods and areas of intervention for European policymakers and AI researchers in turn. Our recommendations urge these stakeholders to *assess* existing capacities and potential opportunities, incentivise the creation of new infrastructures, and develop new approaches to enable society to maximise the potential of AI in the context of climate change, while minimising ethical and environmental drawbacks.

### Recommendations for policymakers

By themselves, comprehensive surveys and conferences appear to be insufficient to gather, document, and analyse all the relevant evidence of AI being used to understand and combat climate change. More needs to be done to monitor and seek positive, climate-focused AI solutions from across sectors, domains, and regions of the world. This would involve deriving best practices and lessons learned from existing projects and identifying opportunities for future initiatives that may be missed without sufficient funding or support. Given the political and economic commitments it has already made, the EU would be an especially suitable sponsor and host of such an initiative. The EU is also in a leading position internationally to disseminate its findings to support action against climate change at a global scale.

**Recommendation 1**: *Incentivise* a world-leading initiative (Observatory) to document evidence of AI being used to combat climate change around the world, derive best practices and lessons learned, document how the values fairness, autonomy and privacy are safeguarded, and disseminate the findings among researchers, policymakers and the public.

Another challenge concerns the ability to share the necessary resources for developing robust AI systems. This includes the best practices and lessons learned to be collected by the initiatives proposed in Recommendation 1 but, crucially, it also extends to data. The effectiveness of AI systems rests in large part on the size and quality of available datasets used to train these systems.

The recent European Strategy for Data notes that a current lack of data hinders the use of data for the public good, underlining the Commission’s support for establishing a new Common European Green Deal “data space [to] support … the Green Deal priority actions on climate change” (European Commission 2020b). This may in turn require legislative and regulatory steps to facilitate business-to-business and business-to-government data sharing. The document also notes the need to “assess what measures are necessary to establish data pools for data analysis and machine learning”. The issue for the climate change data space is not simply to open the floodgates to data sharing, but also to ensure that the data that is shared is high-quality, accurate, and relevant to the problem at hand. In short, this is not just a question of collation but also of curation. The steps outlined so far to ensure that the specific requirements of climate change research are served by the data space are moving in this direction.

The European Strategy for Data argues that “data spaces should foster an ecosystem (of companies, civil society and individuals) creating new products and services based on more accessible data”. In the case of climate change, organisations (particularly in the private sector) may need further encouragement to develop AI-based solutions that are not “products and services” per se, but rather focused efforts to tackle climate-related issues, with or without a profit incentive, and potentially in partnership with public and non-profit groups. Therefore, the Commission could play a more front-footed role in stimulating these efforts or “challenges”, as it has already sought to do in the context of business-to-government data sharing for the public interest more generally (European Commission 2020f).

**Recommendation 2:** *Develop* standards of quality, accuracy, privacy, relevance and interoperability for data to be included in the forthcoming Common European Green Deal data space; identify aspects of climate action for which more data would be most beneficial; and explore, in consultation with domain experts and civil society organisations, how this data could be pooled in a common global climate data space.

**Recommendation 3:** *Incentivise* collaborations between data providers and technical experts in the private sector with domain experts from civil society, in the form of “challenges”, to ensure that the data in the Common European Green Deal data space is utilised effectively against climate change.

As Sect. 4 makes clear, there has been considerable investment—both fiscal and political—to harness the twin ecological and digital transitions to create a more sustainable and prosperous EU. If done right, using AI in the fight against climate change is an ideal point of synthesis for these objectives. Therefore, to build on the previous recommendations, we also recommend that the European Commission earmarks a proportion of the recently announced Recovery Fund to support efforts to develop AI that tackles climate change in the ways identified through the proposals in Recommendation 1. Per the recent agreement between the Commission, the Parliament and European leaders, a considerable proportion (30%) of the Fund will be “dedicated to fighting climate change”, and it is separately stated that more than 50% of the overall fund will support modernisation related, to *inter alia*, “fair climate and digital transitions”. Thus, there is ample scope to invest a substantial proportion of this fund to leveraging AI-based responses to climate change, building on opportunities identified in Recommendations 1–3.

**Recommendation 4:** *Incentivise the development of* sustainable, scalable responses to climate change that incorporate AI technology, drawing on earmarked Recovery Fund resources.

It is important to ensure that all EU-funded and supported climate change research and innovation that uses AI follow steps to prevent bias and discrimination. This should take the form of protocols, auditing, and best practices tailored to this particular research context. In particular, large-scale initiatives such as the Destination Earth project ought to be designed with great care to prevent biases and discrepancies from arising in the so-called “digital twin” that will be created.

At the same time, transparency of purposes—clarifying for what an AI system is being optimised—may help to protect human autonomy. To this end, it may not be enough to make available information about how systems are optimised, but it may also be necessary to give affected stakeholders the opportunity to question, and even contest, the optimisation parameters that are set for a given system, depending on the context. Ensuring that these mechanisms of explanation and contestation are reliable and reproducible is likely to require access to the relevant data and initial conditions and parameter settings that were used for training algorithms.

**Recommendation 5**: *Develop* mechanisms for ethical auditing of AI systems deployed in high-stake climate change contexts, where personal data may be used and/or individual behaviour may be affected. *Ensure* that clear, accessible statements regarding what metrics AI systems are optimised for, and why this is justified, are made available prior to the deployment of these systems. The possibility for affected stakeholders to question and contest system design and outcomes should also be guaranteed.

Policymakers also have an important role to play in equalising access to compute, developing efficient deep learning, and making AI research that is compute-intensive more accessible and affordable. For example, researchers in the US have suggested nationalising cloud infrastructure to provide more researchers with the ability to work without bearing exorbitant costs (Etchemendy and Li 2020). A European equivalent of the “National Research Cloud” could enable the EU to establish a long-term infrastructure that enables more European researchers to compete on a global scale, while also ensuring that research occurs on an efficient and sustainable European platform.^24^

The reported and estimated decrease (by 30%) of EU-based data centres (EEA 2020) is largely due to efforts by EU member states to increase the share of renewable energies in power generation (European Commission 2020a). The CO_2_ emissions stemming from national power generation across EU member states have been decreasing, albeit emission rates differ significantly between different member states. For example, power generation in Estonia emits over 9 times more CO_2_ than in Slovakia (EEA 2020).

**Recommendation 6:** *Develop* greener, smarter and cheaper data infrastructure (e.g., European research data centres) for researchers and universities across the EU.

Given the EU’s increasing interest and investments in AI (Stix 2019), it is also important that the AI sector is considered specifically when formulating environmental policies. Both in research or production settings, AI requires increasingly specialised hardware and services that should be considered in any long-term environmental strategies.

**Recommendation 7:** *Assess* AI and its underlying infrastructure (e.g., data centres) when formulating energy management and carbon mitigation strategies to ensure that the European AI sector becomes sustainable as well as uniquely competitive.

Carbon labels and similar standards can benefit from receiving the endorsement of policymakers and even be required within the EU. Policymakers are key to ensuring that the field of AI research becomes more transparent when it comes to energy consumption and carbon emissions.

**Recommendation 8:** *Develop* carbon assessment and disclosure standards for AI to help the field align on metrics, increase research transparency, and communicate carbon footprints effectively via methods such as adding carbon labels to AI-based technologies and models listed in online libraries, journals, and leaderboards.

These labels would allow researchers and developers to make environmentally informed decisions when choosing components (e.g. model, hardware and cloud provider) for their work. For example, borrowing directly from The Carbon Trust’s (2020) “product carbon footprint labels”, the following labels could be adapted to AI research and distributed in a similar fashion to ACM labels:

- Lower CO_2_eq—indicating that the carbon footprint of a model/product is significantly lower carbon than the market dominant model/product in its category.
- CO_2_eq measured—indicating that the model/product footprint has been measured in accordance with an internationally recognised standard such as product standards: PAS2050, GHG Product Standard and ISO14067.
- Carbon neutral—indicating that the model/product emissions are offset by the issuing organisation.

More informed discussions about the necessity and timeliness of certain compute-heavy research projects can emerge from these systematic disclosures. For example, if OpenAI’s GPT-3 had been trained on the latest NVIDIA hardware A100, a single training run could have been twice as efficient. AI research projects ought to engage actively with, and communicate, the ecological trade-offs they are making. Even if one may not expect researchers to weigh accurately all the potentially beneficial environmental impact that their research project has or could lead to, such cost–benefit analysis should be considered (Rolnick et al. 2019; Henderson et al. 2020).

Policymakers are also key to ensuring that AI researchers in the EU are able to expand the field of AI research and diverge from conventional assumptions and research practices. Diverse funding will help European researchers to break from technological and normative trends that make it costly for researchers to try new ideas.

**Recommendation 9:** *Incentivise* diverse research agendas by funding and rewarding projects that diverge from the current trend of compute-intensive AI research to explore energy-efficient AI.

Examples of potentially energy-efficient AI strategies include new hardware-software-algorithm combinations, algorithmic progress, symbolic AI, and hybrid symbolic-neural systems (Marcus 2020). Following this recommendation would enable the EU to enhance its potential for the development of AI research, by allowing a diverse pool of researchers from multiple countries to pursue a wide range of research agendas and compete with compute-intensive AI research coming from the US and China.

**Recommendation 10:** *Incentivise* energy-efficient and green research by making EU funding conditional on applicants measuring and reporting their estimated energy consumption and GHG emissions. Funding could fluctuate according to the environmental efforts made (e.g. usage of efficient equipment, usage of renewable electricity, Power Usage Effectiveness of < 1.5).

### Recommendations for AI research stakeholders

The field of AI research stakeholders, which includes (but is not limited to) researchers, laboratories, funding agencies, journal editors, conference organisers, and the managers of open-source ML libraries, can take several immediate steps to ensure that its carbon footprint is properly gauged and kept in check. At the same time, policymakers should play a critical role in ensuring that new reporting standards are set for organisations conducting large scale experiments and that the underlying infrastructure of AI remains environmentally sustainable while supporting innovative AI research in the EU. To this end, we offer recommendations to both stakeholders in both the research and policy domains.

Steps have already been taken to tackle the reproducibility crisis mentioned in Sect. 3.3. For example, within two years of encouraging paper submissions to include source code, NeurIPS reported the number of papers with code going from 50 to 75% of submissions (Gibney 2020). Additionally, standards and tools, like the Association for Computing Machinery’s (ACM) (2020) artifact badging, NeurIPS’s (2020) OpenReview, cKnowledge (Fursin 2020), PapersWithCode (2020), and MLPerf (Reddi et al. 2020), have been established in recent years to promote openness in scientific communication and ensure reproducibility. Similarly, systematic and accurate measurements to evaluate the energy consumption and carbon emissions of AI is needed for research activities. “Plug and play” tools need to be developed to facilitate the reporting of GHG emissions, and research conferences, journals and the community at large can play an important role in normalising the reporting of such data.

Open-source ML libraries, which are often established by private organisations, are essential to AI research. Adding information on the energy consumption, carbon emissions, and training conditions of various models—including hyperparameter sensitivity or algorithm performance against hardware—on these websites can help the field develop its environmental commitment.

**Recommendation 11:** *Develop* conference and journal checklists that include the disclosure of, *inter alia*, energy consumption, computational complexity, and experiments (e.g. number of training runs, and models produced) to align the field on common metrics (Gibney 2020; Schwartz et al. 2019; Henderson et al. 2020).

**Recommendation 12:** *Assess the* carbon footprint of AI models that appear on popular libraries and platforms, such as PyTorch, TensorFlow and Hugging Face, to inform users about their environmental costs.

These recommendations aim to normalise the disclosure of information pertaining to AI’s carbon footprint as well as to help researchers and organisations select research tools based on environmental considerations. Online AI courses, ML libraries, journals and conferences can take actions to collect and display more information regarding the energy consumption and GHG emissions of AI. These recommendations aim to enable researchers to monitor and report systematically their AI projects’ carbon footprints using ready-made tools such as Lacoste et al.’s calculator (2019) or Henderson et al.’s framework (2020).

Increasing research on energy efficient computing and efficient AI is an important component to ensure that AI’s carbon footprint is controlled in the long run. And the promotion of efficiency metrics and research may need to come from the field itself. For example, the low-power image recognition challenge was created to define a common metric to compare image recognition results, accounting for energy efficiency and accuracy (Gauen et al. 2017). Similarly, Stanford University’s DAWNbench benchmark was created in response to the field’s hyper focus on accuracy metrics (Coleman et al. 2019b). The benchmark offers a “reference set of common deep learning workloads for quantifying training time, training cost, inference latency, different optimisation strategies, model architectures, software frameworks, clouds, and hardware” (Coleman et al. 2019a).

**Recommendation 13:** *Incentivise* the development of efficiency metrics for AI research and development (including model training) by promoting efficiency improvements and objectives in journals, conferences and challenges.

Note that key organisations such as the ACM, IEEE, NeurIPS and ICML, among others, would be instrumental in normalising efficiency metrics or publication requirements such as the one outlined in Recommendation 6. The normalisation of such metrics and requirements can bring more researchers to actively seek energy-efficient approaches to research and development, such as by changing their region of compute or cloud provider, or by opting to run intense calculations during times of excess electricity generation capacity (e.g. at night).

## Conclusion

In this article, we have analysed the beneficial impact that AI can have in the fight against climate change, the ethical challenges encountered in this process, and the computational intensity that the development of AI requires, which introduces different challenges relating to energy consumption and GHG emissions. Benefits and risks are distinct yet intertwined. This is why we agree with Floridi and Nobre (2020) and see the use of AI to fight climate change as a leading example of

“a new marriage between the Green of our habitats—natural, synthetic and artificial, from the biosphere to the infosphere, from urban environments to economic, social, and political circumstances—and the Blue of our digital technologies, from mobile phones to social platforms, from the Internet of Things to Big Data, from AI to future quantum computing”.

In this marriage, some risks, such as AI’s carbon footprint, are not entirely avoidable, but they can certainly be minimised, to deliver the best strategies against climate change. This is why the right policies are key to harness the opportunities while ensuring that the risks are adequately assessed and minimised, as much as possible.

Harnessing the positive and mitigating the negative impact of AI on the environment is achievable with the support of robust policymaking and of key stakeholders. The formula of “Green & Blue” has never been more central to the European policymaking agenda, and the Recommendations outlined in this article can serve as a “Green & Blue-print” for a more sustainable society and a healthier biosphere. By shedding light on the use of AI to counter climate change and offering recommendations to make this use of AI ethically sound and sustainable, this article aims to inform EU policy strategy for the ‘twin transitions’ and help ensure that the marriage between the Green and the Blue is a success that leads to a better society and a healthier planet.

## Abbreviations

ACM: Association for Computing Machinery
AI: Artificial intelligence
ASICs: Application-specific integrated circuits
AWS: Amazon web services
Compute: Computing power
CCAI: Climate change AI
CPU: Central processing unit
CO_2_eq: Carbon dioxide equivalent
DL: Deep learning
EU: European Union
ELLIS: European Lab for Learning and Intelligent Systems
FLOPS: Floating point operations per second
FPGAs: Field programmable gate arrays
GDP: Gross domestic product
GDPR: General data protection regulation
GHG: Greenhouse gas
GPU: Graphics processing unit
ICML: International Conference on Machine Learning
ICT: Information Communication Technologies
IJCAI: International Joint Conferences on Artificial Intelligence
IPCC: Intergovernmental Panel on Climate Change
ML: Machine learning
NeurIPS: Conference on Neural Information Processing Systems
NLP: Natural language processing
RL: Reinforcement learning
SOTA: State-of-the-art
SDGs: Sustainable development goals
TPU: Tensor processing unit
UN: United Nations

## Footnotes

1 https://ellis.eu/programs/machine-learning-for-earth-and-climate-sciences.

2 https://copernicus-masters.com/prize/esa-challenge/#.

3 https://imiracli.web.ox.ac.uk.

4 https://www.uv.es/uvweb/uv-news/en/news/ai-understanding-modelling-earth-system-international-research-team-co-led-university-valencia-wins-erc-synergy-grant-1285973304159/Novetat.html.

5 https://www.exeter.ac.uk/research/environmental-intelligence/.

6 https://ai4er-cdt.esc.cam.ac.uk.

7 Search of Cordis research project database conducted 30th November 2020 of Projects with search string [('climate change' OR ‘global warming’) AND ('artificial intelligence' OR 'machine learning')], (*n* = 122).

8 https://www.microsoft.com/en-us/ai/ai-for-earth-partners.

9 https://microsoft.github.io/AIforEarth-Grantees/.

10 https://ai.google/social-good/impact-challenge/.

11 https://www.elementai.com/ai-for-climate.

12 https://www.sbs.ox.ac.uk/research/centres-and-initiatives/oxford-initiative-aisdgs (last accessed 11 Aug 2021).

13 https://www.itu.int/en/ITU-T/AI/Pages/ai-repository.aspx.

14 http://www.ai-for-sdgs.academy/topics#13%20Climate%20Action.

15 Machine learning is commonly considered to be a subset of the wider set of technological systems that fall under the heading of artificial intelligence. Rolnick et al.’s usage of “machine learning”, however, is quite inclusive, capturing a broad array of examples. Rather than split definitional hairs, the evidence assembled is treated here as a comprehensive overview of the ways in which artificial intelligence per se can be used to combat climate change.

16 https://www.climatechange.ai/about.

17 A new standard (L.1470) set out by the ITU was recently developed to keep the ICT industry in line with the Paris Agreement and reduce GHG emissions by 45% from 2020 to 2030 (ITU 2020).

18 Over the past two decades, the number of data-driven AI conferences and publications has grown dramatically (Perrault et al. 2019). The volume of peer-reviewed AI papers increased by more than 300% from 1998 to 2018, while the number of publications on “Machine Learning” on the open-access archive “ArXiv” has doubled every 15 months since 2015 (Perrault et al. 2019). This growth is also fuelled by an increasing amount of publications originating from the private sector—big technology companies in particular—with their seemingly limitless resources to conduct experiments (Perrault et al. 2019, 17; Lohr 2019; Ahmed and Wahed 2020).

19 It is important to also include field programmable gate arrays (FPGAs) and application-specific integrated circuits (ASICs) like tensor processing units (TPUs) to this framework.

20 The 28 TFLOPS is assumed here based of NVIDIA’s advertisement of the V100 performance as well as on Microsoft’s DeepSpeed and ZeRO-2 performance results for training + 100-billion-parameter models using V100 GPUs (NVIDIA 2018; Rangan and Junhua 2020).

21 Using a different set of assumptions and a methodology similar to that of Henderson et al. (2020), a group of researchers from the University of Copenhagen have estimated the training run would emit up to 84,738.48 kg CO2eq in the US (region not specified) (Anthony et al. 2020). This highlights the importance of disclosing enough information in research publications.

22 It is also worth noting the carbon footprint associated with the steep growth in attendance at AI conferences. The Stanford AI Index reports that in 2019, NeurIPS had an increase of 41% in attendance over 2018 and over 800% relative to 2012 (Perrault et al. 2019). Other conferences such as the AAAI and CVPR are also seeing an annual attendance growth of around 30% (Perrault et al. 2019). These generate a non-negligible amount of carbon emissions (Perrault et al. 2019; Henderson et al. 2020). Using the attendance of the top 10 AI conferences in 2019, Henderson et al. (2020) estimate that around 34,449,597 kg of CO_2_eq were emitted from these conferences alone.

23 Once-for-all networks support “diverse architectural settings by decoupling training and search”, thus reducing GPU usage and carbon emissions (Cai et al. 2020, 1).

24 Such a project would present clear synergies with the EU’s landmark cloud infrastructure project, Gaia-X, which seeks to develop a common data infrastructure in Europe (GAIA-X 2020).
